# Fractionated Seminal Plasma of Boar Ejaculates Analyzed by LC–MS/MS: Its Effects on Post-Thaw Semen Quality

**DOI:** 10.3390/genes12101574

**Published:** 2021-10-02

**Authors:** Leyland Fraser, Karolina Wasilewska-Sakowska, Łukasz Zasiadczyk, Elżbieta Piątkowska, Krzysztof Karpiesiuk

**Affiliations:** 1 Department of Animal Biochemistry and Biotechnology, Faculty of Animal Bioengineering, University of Warmia and Mazury in Olsztyn, 10-719 Olsztyn, Poland; kwasilewska@cyfroweszkoly.pl (K.W.-S.); lukasz.zasiadczyk@uwm.edu.pl (Ł.Z.); 2 Laboratory of Mass Spectrometry Research Centre EIT+, Stabłowicka 147, 54-066 Wrocław, Poland; elzbieta.piatkowska@eitplus.pl; 3 Department of Pig Breeding, Faculty of Animal Bioengineering, University of Warmia and Mazury in Olsztyn, 10-719 Olsztyn, Poland; krzysztof.karpiesiuk@uwm.edu.pl

**Keywords:** boar, seminal plasma proteins, spermatozoa, LC–MS/MS, cryopreservation

## Abstract

This study aimed to characterize the protein composition of fractionated seminal plasma (SP) by liquid chromatography mass spectrometry (LC–MS/MS) analysis and investigate its effects on survival of frozen-thaw (FT) boar spermatozoa following storage. Seminal plasma (SP) was fractionated by gel filtration chromatography to give two fractions, SP1 with more than 40 kDa (>40 kDa) and SP2 with less than 40 kDa (<40 kDa). SP1 and SP2 were subjected to LC–MS/MS and bioinformatics analysis. Following cryopreservation, FT boar semen (*n* = 7) was thawed in Beltsville Thawing Solution (BTS), BTS + SP1 or BTS + SP2, stored at different periods and subjected to post-thaw (PT) quality assessment. A total of 52 and 22 abundant proteins were detected in SP1 and SP2, respectively. FN1, ANGPTL1, and KIF15 proteins were more abundance in SP1, whereas a high abundance of spermadhesins (PSP-I and PSP-II) was detected in SP2. Proteins of the fractionated SP were involved in various biological processes, such as cell motility and signal transduction. The dominant pathway of SP1 proteins was the apelin signaling pathway (GNA13, MEF2D, SPHK2, and MEF2C), whereas a pathway related to lysosome (CTSH, CTSB, and NPC2) was mainly represented by SP2 proteins. In most of the boars, significantly higher motility characteristics, membrane integrity, and viability were observed in FT spermatozoa exposed to SP1 or SP2 compared with BTS. The results of our study confirm that a combination of several proteins from the fractionated SP exerted beneficial effects on the sperm membrane, resulting in improved quality characteristics following PT storage.

## 1. Introduction

The limited use of frozen–thawed boar semen is mainly due to the substantially reduced fertilizing ability of frozen–thawed (FT) spermatozoa [1,2]. Many efforts have been made to improve the freezing protocol of boar semen, including focusing on the identification of seminal plasma (SP) markers associated with semen freezability [3,4,5,6]. Moreover, boar SP proteins were suggested to be potential markers for fertility in artificial insemination (AI) of boars [7,8]. Given the importance of SP proteins, functional proteomics has been used as a new tool to identify markers associated with semen fertility or sperm cryo-tolerance [9,10,11,12].

Boar SP comprises a diverse set of secretions, originating from the accessory sex glands, epididymis, and testis [9]. Even though most boar SP proteins in sperm cryo-survival have not as yet been fully elucidated [13], evidence shows the beneficial effects of specific SP components on post-thaw (PT) semen quality and fertility [14,15,16,17,18,19]. Additionally, components of the SP were shown to prolong sperm cryo-survival [16,17,20] and could inhibit or reverse capacitation of spermatozoa during cooling [21] or cryopreservation [22]. Evidence has shown that differences in the SP compositions among boars and ejaculates could affect PT semen quality [14,15,17,18]. Moreover, marked differences in the SP proteome profiles are associated with high-fertility and low-fertility boars, and suggest that SP proteins can be used as biomarkers to select high-fertility boars [8,23]. It is noteworthy that components of the whole SP were shown to compromise sperm cryo-survival [15,17,24]. Studies have demonstrated that SP of the sperm-rich fraction (SRF) provide better sperm cryo-survival than the post-SRF fraction [13,17,22]. However, even though progress has been made in the study of the SP proteome, the physiological role of some of its components, in relation to its interactions with the sperm membrane, is still not fully understood. It is well accepted that specific SP components with low- and high-molecular weights are adsorbed onto the surface of ejaculated spermatozoa during their transport in the reproductive tract to maintain the stability of the sperm membranes [9,25,26]. Due to the limited sensitivity of two-dimensional electrophoresis (2DE), and the possibility that highly-abundant SP components could mask the detection of a low-abundance of proteins of the SP [11,26], we have considered that chromatographic methods, in conjunction with liquid chromatography mass spectrometry (LC–MS/MS) analysis, are useful approaches to detect potential SP proteins that are implicated in sperm cryo-survival.

Using gel filtration chromatography of fractionated SP of boar ejaculates, we have demonstrated that the incorporated two SP fractions with molecular weights, more than 40 kDa (>40 kDa, SP1) or less than 40 kDa (<40 kDa, SP2) in the freezing protocol, resulted in improved cryo-survival of boar spermatozoa [27,28]. The objectives of this study were to (i) characterize the protein composition of the fractionated SP (SP1 and SP2) by LC–MS/MS analysis, and (ii) investigate the effect of supplementation of SP1 or SP2 at post-thaw on the quality characteristics (motility parameters, membrane integrity, and viability) of semen from individual boars.

## 2. Materials and Methods

### 2.1. Chemicals and Media

All chemicals were bought from Sigma-Aldrich Chemicals Company (St. Louis, MO, USA), unless otherwise stated. The fluorescent probes, 5,5′,6,6′-tetrachloro-1,1′,3,3′-tetraethylbenzimidazolylcarbocyanine iodide (JC-1), SYBR-14 (Live/Dead Sperm Viability Kit) and propidium iodide (PI) were purchased from Molecular Probes (Eugene, OR, USA).

### 2.2. Animal and Semen Collections

Ejaculates were collected from seven sexually mature Polish large white (PLW) boars, designated (aged 1.5 to 2 years) using the gloved hand technique. Ejaculates were collected from four boars stationed at the Cryopreservation Laboratory (Faculty of Animal Bioengineering, University of Warmia and Mazury in Olsztyn, Poland), and three boars at the Artificial Insemination Station in Ciechanów (Poland). A total of 100 mL ejaculates, comprising the sperm-rich fraction (SRF) and a portion of the post-SRF, were collected from each boar for SP analysis [27]. For the cryopreservation procedure, a total of four or seven sperm-rich fractions (SRFs) were collected from the boars. All ejaculates and the SRFs were collected in pre-warmed graded cylinders during the autumn–winter period (from October through March). At collection, the gel portion was removed, using double gauze. The boars were fed with the same commercial porcine ration throughout the entire experimental period. Water was available ad libitum. The SRF of each boar had more than 70% total sperm motility (TMOT) and less than 15% spermatozoa with abnormal morphology. Sperm concentration was determined, using a Bürker counting chamber (Equimed Medical Instruments, Kraków, Poland). The study was divided into two experiments: experiment 1 comprised liquid chromatography–tandem mass spectrometry (LC–MS/MS) analysis of the fractionated SP. Experiment II included semen cryopreservation procedure, PT semen treatment, and analysis. Approval of the Local Ethics Committee for experiments on boars (semen collections) has not been required since 15 January 2015.

### 2.3. Experiment I

#### 2.3.1. Sample Preparation and Digestion for Nano LC–MS/MS Analysis

All chemicals were purchased from Sigma-Aldrich Chemicals Company (St. Louis, MO, USA), unless otherwise stated. Following the measurements of the protein content [29], the SP was subjected to gel filtration chromatography (Fast Protein Liquid Chromatography, FPLC) on Sephacryl S-200 HR HiPrep 16/60 column (Amersham-Pharmacia, Biotech) to provide two chromatographic fractions: SP1 (>40 kDa) and SP (< 40 kDa), as described in a previous study [27].

Prior to analysis, the fractionated samples of SP1 or SP2 were pooled. Samples were processed with the Filter Aided Sample Preparation (FASP), according to a previously described study [30]. Briefly, 100 µg of total protein were added to a Tris-urea buffer containing 8 M urea, 0.1 M Tris/HCl (pH 8.5), and 0.1 M dithiothreitol (DTT), and the mixture was incubated for 30 min at 60 °C. The protein samples were loaded onto Microcon 30 kDa molecular weight cut-off centrifugal filters (Merck, Millipore, KGaA, Darmstadt, Germany) and centrifuged for 15 min at 15,000× *g* at room temperature. The filter membranes were washed twice with the Tris-urea buffer, followed by alkylation, with 50 mM iodoacetamide for 20 min in the dark. Following 2× more washing with Tris-urea buffer filter, the membranes were further washed 2× with 25 mM ammonium bicarbonate to remove the detergents. The proteins on the filter membranes were subjected to digestion with trypsin (1:5 trypsin to 25 mM ammonium bicarbonate ratio) overnight at 37 °C. The digested samples were eluted with ammonium bicarbonate, de-salted with C18 zip tips (Millipore), and dried in a vacuum centrifuge, before being re-suspended in 0.1% formic acid for further analysis.

#### 2.3.2. Protein Identification and Quantification

One microgram of peptide extract was injected into the nano-LC (nLC-1000 nano flow) HPLC system (Thermo Fisher Scientific) coupled via a nano-electrospray ion source to Orbitrap Elite (Thermo Fisher Scientific) FTMS (Fourier transform-based mass spectrometry). Chromatographic analyses were performed using an analytical column (Acclaim PepMap 100 C18, 50 cm, 75 µM ID, 3 µM) with 120 min gradient. Two different buffer systems were used: buffer A (0.1% formic acid in water, *v*/*v*) and buffer B (90% acetonitrile and 0.1% formic acid in H_2_O) with a gradient of peptide separation from 0 to 100 min 45% B, and a flow rate of 300 µL/min followed by column washing and the equilibration steps. The MS data were acquired with a Top15 data-dependent MS/MS scan method in the range of 100–2000 *m*/*z* and resolutions at 120,000 for MS and 15,000 for MS/MS spectra (at *m*/*z* 400). Fragmentation of peptides was performed using the higher-energy collisional dissociation (HCD) mode (normalized collision energy of 35 eV) and dynamic exclusion set to 15 s. The LC–MS/MS raw data were analyzed with the MaxQuant software (v.1.4.1.2) for peak detection and quantification [31]. The LC–MS/MS spectra were searched against the UniProtKB/Swiss-Prot database for *Sus scrofa* for protein identification and quantification using the Andromeda search engine [32]. Label-free quantification (LFQ) procedure was performed to identify peptides in order to quantify protein abundance in the profiles represented by fractionated SP (SP1 and SP2) from the pooled samples of the boars.

#### 2.3.3. Gene Ontology (GO) Analysis, KEGG, and REAC Pathways

Functional enrichment of the abundant proteins of SP1 and SP2 in Gene Ontology (GO) categories: molecular function (GO:MF), biological process (GO:BP), and cellular component (GO:CC), was performed with the on line tool, the g:Profiler (accessed on 14 April 2021; https://biit.cs.ut.ee/gprofiler/gost) [33]. We analyzed the significance of SP1 and SP2 proteins in the Kyoto Encyclopedia of Genes and Genomes (KEGG) and Reactome (REAC) biological pathways with the functional enrichment tool provided by the g:Profiler tool. The version of the g:Profiler was e103_eg50_p15_eadf141. Annotations were performed with the *Sus scrofa* database, using the false discovery rate (FDR) of the Benjamini–Hochberg (BH) method for the significance threshold. The user threshold was at 0.05. Heatmaps, GO plots, and Bubble plots for KEGG pathways were performed using GraphPad Prism software (GraphPad Prism v.9.2.0. for Windows, GraphPad Prism software, San Diego, CA, USA, accessed on 15 July 2021; www.graphpad.com). The Panther Classification system (v.16) was used to identify the protein classes of the SP1 and SP2 [34].

To further investigate the functional roles of the abundant proteins of SP1 and SP2 in boar ejaculates, we performed the protein–protein interaction (PPI) networks using the FunRich software tool v.3.1.4. (accessed on 2 December 2020; http://www.funrich.org). The FunRich tool for interactions [35] was customized using the *Sus scrofa* database in UniProtKB (https://www.uniprot.org/uniprot/?query=taxonomy:9823 accessed on 11 September 2021). The statistical significance of enriched terms was performed by a hypergeometric distribution test, and the FDR method was implemented to correct for multiple testing.

### 2.4. Experiment II: Semen Processing Procedure and Quality Assessment

#### 2.4.1. Semen Cryopreservation

The SRFs, following analyses, were diluted in Beltsville Thawing Solution (BTS) and the extended samples were processed according to a previously described cryopreservation protocol, using 5% lyophilized lipoprotein fractions of ostrich egg yolk, LPFo [27,36,37]. Following centrifugation (800× *g* for 10 min) of the cooled semen, the sperm pellets were re-suspended in an LPFo-extender containing 11% lactose (lactose-LPFo extender). The LPFo-extended semen was cooled to 5 °C for 2 h, and further diluted (2:1) with a freezing extender containing 89.5 mL lactose-LPFo extender, 9 mL glycerol (*v*/*v*), and 1.5 mL Orvus Es Paste. The final sperm concentration was approximately 500 × 10^6^ spermatozoa/mL. Semen samples were frozen in a controlled programmable freezer (Ice Cube 14 M, SY-LAB, Austria) and stored in liquid nitrogen, prior to thawing in a water bath for 60 sec at 50 °C.

#### 2.4.2. Post-Thaw Semen Treatment

Following thawing, the samples (500× 10^6^ spermatozoa/mL) of each boar were divided into three portions, and each portion was diluted with BTS, BTS + SP1 (1:1) and BTS + SP2 (1:1) [28]. For each boar, FT spermatozoa were held in homologous SP, supplemented with BTS, and incubated for 15 min (0 h), 60 min (1 h), 120 min (2 h), and 180 min (3 h) at 37 °C, prior to analysis of motility characteristics, membrane integrity, and viability. The final concentration after dilution was 50 × 10^6^ spermatozoa/mL. Fractionated SP of each boar was used for PT storage. Sperm quality was analyzed in fresh, pre-freeze semen and PT semen.

#### 2.4.3. Semen Quality Analysis

##### Motility Parameters Analyzed by the Computer-Assisted Sperm Analysis (CASA) System

Sperm samples (5 µL) were placed on a pre-warmed Makler counting chamber and examined at 37 °C, using the computer-assisted sperm analysis (CASA) system (HTR-IVOS 12.3, Hamilton Thorne Biosciences, MA, USA). The sperm parameters analyzed by the CASA system included total motility (TMOT, %), progressive motility (PMOT, %), and rapid movement (%). A minimum of five fields per sample were assessed, with approximately 200 spermatozoa per field, using the CASA sperm parameters described in a previous study [38].

##### Membrane Integrity Assessment

The percentages of spermatozoa with high mitochondrial membrane potential (MMP) were assessed with dual fluorescent probes, JC-1 with propidium iodide (PI) (Molecular Probes, Eugene, USA), according to previously described studies [39,40]. Briefly, JC-1 solution was added to sperm samples (30 × 10^6^ spermatozoa/mL in HEPES saline medium) and samples were incubated for 15 min at 37 °C, prior to PI addition. Stained slides were examined under a fluorescence microscope (Olympus CH 30, Tokyo, Japan) at 600× magnification.

The percentages of membrane-intact spermatozoa were evaluated by double florescent staining with SYBR-14 and PI, using the Live/Dead Sperm Viability Kit (Molecular Probes, Eugene, OR, USA), according to a previously described method [41]. Briefly, sperm samples (30 × 10^6^ spermatozoa/mL) were incubated in 1 mM SYBR-14 solution and 2.4 µM PI for 10 min at 37 °C. Aliquots of the stained sperm cells were examined at 600× magnification under a fluorescence microscope (Olympus CH 30). A minimum of 100 cells per slide were examined and classified as membrane-intact and membrane-damaged spermatozoa. Each slide was analyzed in duplicate.

Sperm acrosome integrity (%) was evaluated using fluorescein isothiocyanate-labeled peanut (arachis hypogaea) agglutinin (FITC-PNA) staining with PI, according to a previously described method [42], with some modifications [27]. Aliquots of the sperm samples (3 × 10^6^ spermatozoa/mL in HEPES saline medium were mixed with FITC-PNA solution (2 mg FITC-PNA in 1 mL PBS), and were incubated for 10 min at 37 °C. Following incubation, 10 µL of PI (1 mg PI in PBS) were added and incubated for 5 min at 37 °C. The stained sperm cells (10 µL) were spread on a pre-cleaned microscopic slide, mounted with 5 µL of Prolong^®^ Diamond Antifade Mountant (Molecular Probes, Eugene, OR, USA), and covered with a coverslip. At least 100 spermatozoa in each duplicate were assessed at 600× magnification under a fluorescence microscope.

##### Viability Assessment

The percentages of viable and plasma membrane apoptotic-like changes in spermatozoa were assessed using the Vybrant Apoptosis Assay Kit #4 (Molecular Probes, Inc., Eugene, USA), according to a previously described method [37]. Following incubation of sperm suspension (4 × 10^6^ spermatozoa/mL) in YO-PRO-1 (100 µM) and PI (2 μM) solutions for 15 min at 37 °C, aliquots of the stained sperm cells were examined at 600× magnification under a fluorescence microscope (Olympus CH 30). A minimum of 100 cells per slide were examined in each aliquot, and three sub-populations were identified: viable spermatozoa categorized as negative for both YO-PRO-1 and PI (YO-PRO-1^−^/PI^−^), plasma membrane apoptotic-like changes in spermatozoa (moribund spermatozoa) were categorized as positive for YO-PRO-1^+^ but negative for PI^−^ (YO-PRO-1^+^/PI^−^), and dead spermatozoa, which were positive for both YO-PRO-1 and PI (YO-PRO-1^+^/PI^+^).

### 2.5. Statistical Analysis

The one-way analysis of variance (ANOVA) assumption was evaluated using the Shapiro–Wilk W-test to examine the normality of the data distribution. Data were examined by repeated measure ANOVA, using the general linear model (GLM) procedure from Statistica software package, version 12.5 (StatSoft Incorporation, Tulsa OK., USA). A 7 × 3 × 4 factorial design was performed to determine if boar, treatment (BTS, SP1, and SP2), PT storage time (0, 1, 2, and 3 h), or their interactions affected motility characteristics of FT spermatozoa. The effects of boar, treatment, and storage time (0 and 2 h), and their interactions on membrane integrity and viability of FT spermatozoa, were analyzed under a 7 × 3 × 2 factorial design. All results are expressed as the mean ± standard error of the mean (S.E.M). Significant differences among treatment groups were compared using the Newman–Keuls post *hoc test*. The values were considered to differ significantly at *p* < 0.05.

## 3. Results

### 3.1. Analysis of Fractionated SP Proteins

#### 3.1.1. LC–MS/MS Analysis

**Figure 1:**
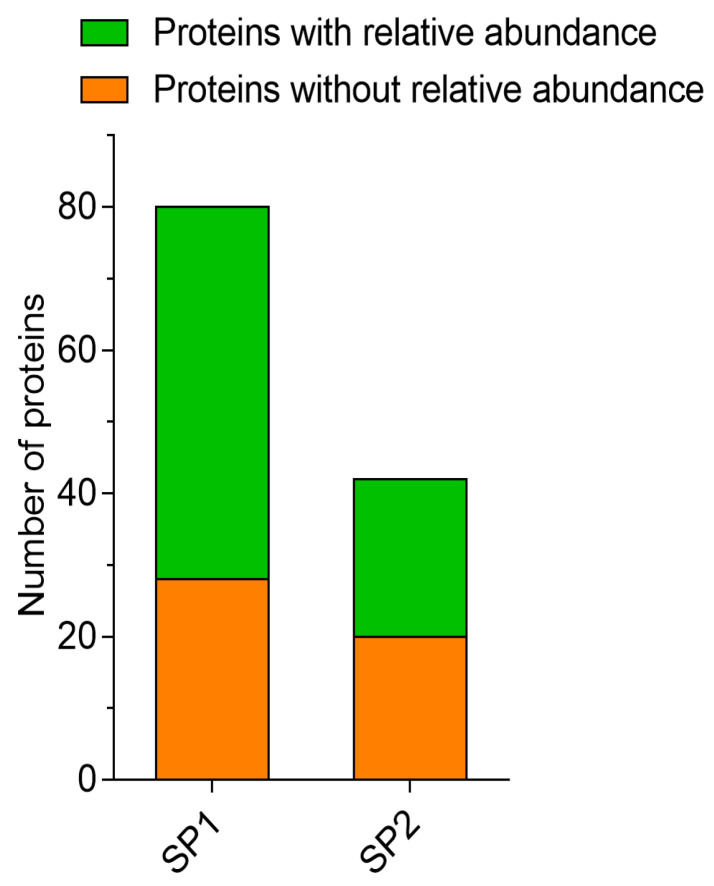
Relative abundance of proteins in fractionated seminal plasma (SP) of boar ejaculates. SP1—seminal plasma 1; SP2—seminal plasma 2.

**Figure 2:**
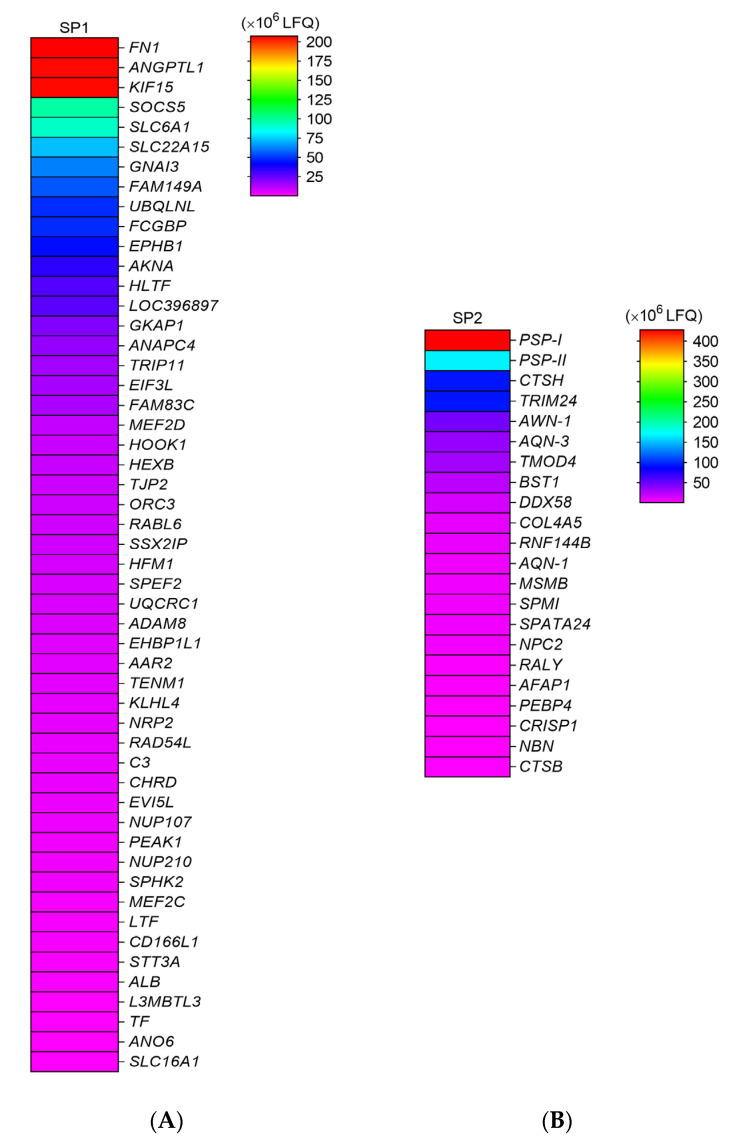
Heatmaps showing relative abundance of proteins in fractionated seminal plasma (SP) of boar ejaculates. (**A**) SP1; (**B**) SP2. Protein abundance was measured by the label-free quantification (LFQ) method.

Using LC–MS/MS analysis, we detected a total of 122 proteins (Supplementary Table S1) in the fractionated SP, but considering proteins with relative abundance, as analyzed by the LFQ method, the total number of abundant proteins were 74, of which 52 (64.1%) and 22 (67.5%) proteins were identified in SP1 and SP2 (Figure 1). The characteristics of the proteins detected in SP1 and SP2 are shown in the Supplementary Table S1. Proteins with high abundance in SP1 included fibronectin 1 (FN1), angiopoietin-related protein 1 (ANGPTL1), and kinesin family member 15 (kinesin-like protein KIF 15) (Figure 2A), whereas PSP-I and PSP-II spermadhesins, cathepsin H, and tripartite motif containing 24 (TRIM24) proteins were more abundant in SP2 (Figure 2B).

#### 3.1.2. GO Analysis, KEGG, and REAC Pathways

**Figure 3:**
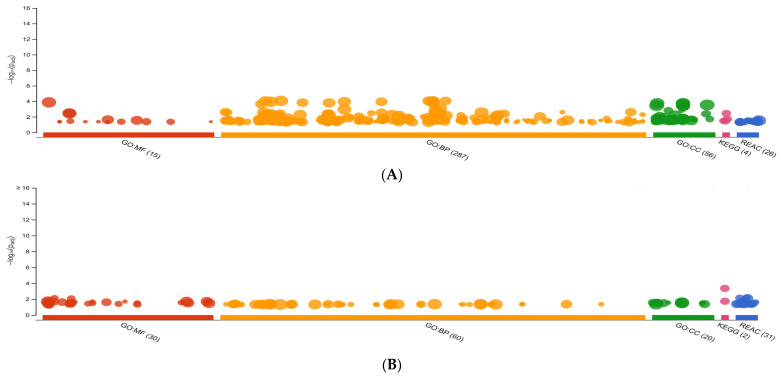
g.GOSt multiquery Manhattan plots showing enrichment analysis of relative abundance of proteins in fractionated seminal plasma (SP) of boar ejaculates. (**A**) SP1 and (**B**) SP2. Gene ontology (GO) terms for molecular function is indicated in red (GO:MF), biological process in orange (GO:BP), and cellular component in green (GO:CC). Kyoto Encyclopedia of Genes and Genomes (KEGG) and Reactome (REAC) enrichment pathway terms are shown in pink and blue colors, respectively.

**Figure 4:**
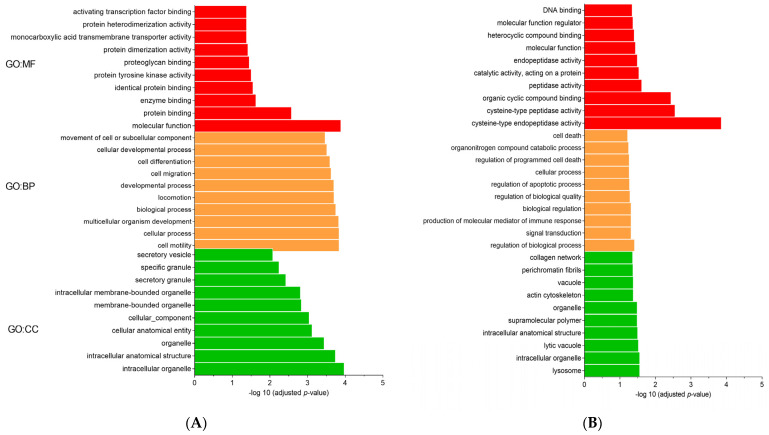
Gene ontology (GO) enrichment of relative abundance of proteins in fractionated seminal plasma (SP) of boar ejaculates. (**A**) SP1 and (**B**) SP2. Ten highly significant GO terms for molecular function (GO:MF), biological process (GO:BP), and cellular component (GO:CC) are presented.

The g.GOSt multiquery Manhattan plots of the GO analysis, and KEGG and REAC pathways of SP1 and SP2 proteins, performed with the KOBAS annotation tool (v.3.0), are shown in Figure 3A,B, respectively. For the SP1 proteins the GO:MF terms were dominated by molecular function and protein binding, such as FN1 and ANGPTL1, whereas the GO:BP and GO:CC terms were mainly represented by cell motility (for example, ADAM8 and PEAK1) and intracellular organelle, such as AKNA and GKAP1, respectively (Figure 4A). Furthermore, for the SP2 proteins the GO:MF term was represented mainly by cysteine-type endopeptidase activity (for example, CTSH and CTSB), while GO:BP and GO:CC terms were mainly represented by regulation of biological process (such as COL4A5 and AFAP1) and lysosome (CTSB and NPC2), respectively (Figure 4B).

**Figure 5:**
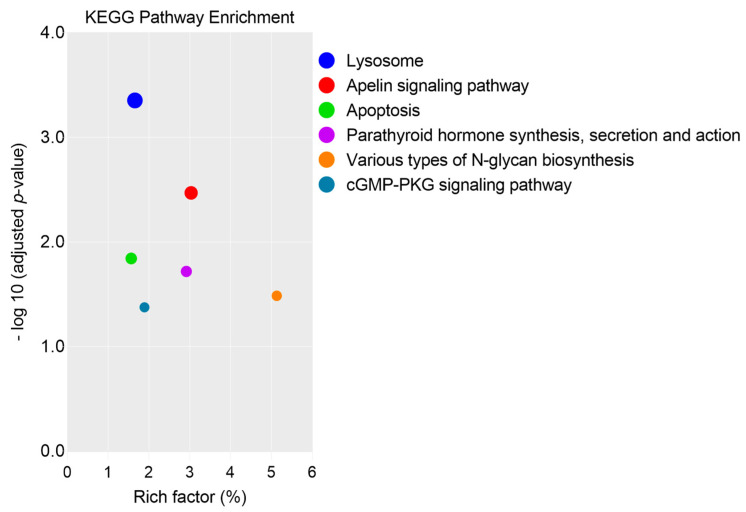
Kyoto Encyclopedia of Genes and Genomes (KEGG) pathway enrichment of proteins in fractionated seminal plasma (SP1 and SP2) of boar ejaculates.

The abundant proteins of SP1 were assigned to four KEGG pathways, such as apelin signaling pathway (GNA13, MEF2D, SPHK2, and MEF2C), parathyroid hormone synthesis, secretion pathway (GNA13, MEF2D, and MEF2C), various types of N-glycan biosynthesis (HEXB and STT3A), and cGMP-PKG signaling pathway (GNA13, MEF2D, and MEF2C), as shown in Figure 5 (Supplementary Table S2A). On the other hand, two KEGG pathways, lysosome (CTSH, CTSB, and NPC2) and apoptosis (CTSH and CTSB) were detected in SP2 (Figure 5, Supplementary Table S3A).

**Figure 6:**
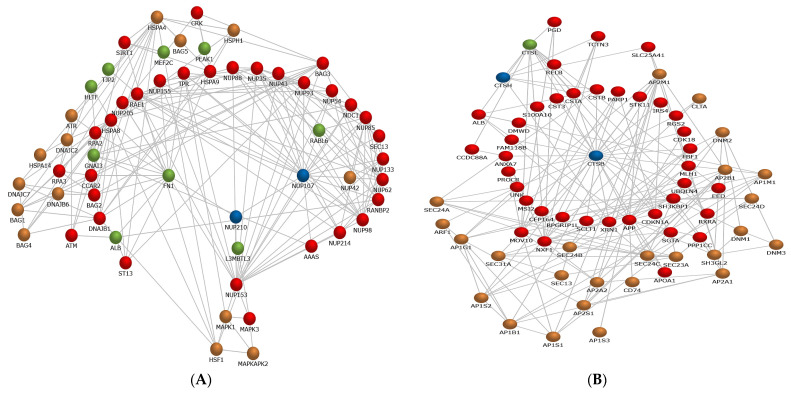
Integration networks of proteins associated with of fractionated seminal plasma (SP1 and SP2). (**A**) Regulation of HSF1-mediated heat shock response for SP1. (**B**) Major histocompatibility (MHC) class II antigen presentation for SP2. Blue nodes indicate the selected protein, while green nodes represent proteins from the neighboring pathway. Red and brown nodes represent interacting proteins of the enriched pathways of existing research. Network interactions were generated from the FunRich software tool v.3.1.4 (2 December 2020; http://www.funrich.org).

The dominant REAC pathway for the abundant proteins of SP1 was represented by “Regulation of HSF1-mediated heat shock response” NUP107 and NUP210) (Figure 6A, Supplementary Table S2B). Likewise, the dominant REAC pathway of SP2 proteins included “Major histocompatibility (MHC) class II antigen presentation” (CTSH and CTSB) (Figure 6B, Supplementary Table S3B). Integrated network of SP1 proteins showed the interactions of eleven proteins (two selected proteins together with nine proteins of the neighboring pathway) were associated with the REAC pathway, “Regulation of HSF1-mediated heat shock response” (Supplementary Table S2B). Regarding SP2, two proteins (two selected proteins together with one protein of the neighboring pathway) were associated with “MHC class II antigen presentation” REAC pathway (Figure 6B, Supplementary Table S3B).

#### 3.1.3. Protein Classes of Fractionated SP

**Figure 7:**
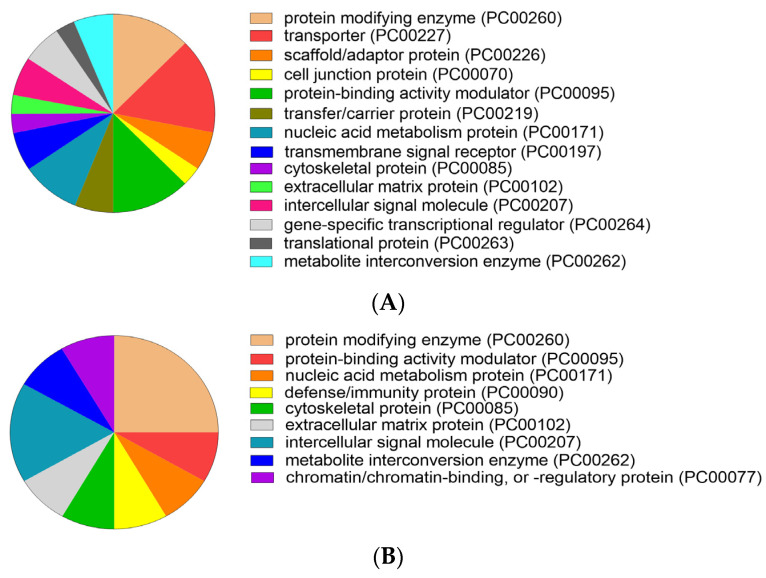
Protein classes in fractionated seminal plasma of boar ejaculates. (**A**) SP1 and (**B**) SP2. The protein class was analyzed by the PANTHER classification system (v.16).

Analysis showed that the abundant proteins of SP1 and SP2 comprised 14 and 9 proteins classes (Figure 7A,B, respectively). The major protein class of the SP1 was transporter (SLC6A1, SLC22A15, NUP107, NUP210, and SLC16A1), representing approximately 15.6% of the total protein class (Figure 7A). Protein modifying enzyme (CTSH, CTSB, and RNF144B) represented approximately 25.0%, was predominant in SP2 (Figure 7B).

### 3.2. Post-Thaw Semen Assessment

There were no significant differences (*p* > 0.05) in the fresh semen parameters among the boars (Table 1). The analysis of variance (ANOVA) revealed that PT sperm motility characteristics, analyzed by the CASA system, were significantly (*p* < 0.001) affected by the boar, treatment, and PT storage (Table 2A). Furthermore, boar × treatment and boar × PT storage interactions had significant effects on sperm TMOT and PMOT (Table 2A). Similarly, boar, treatment, and PT storage significantly (*p* < 0.001) affected most of the analyzed sperm parameters of membrane integrity (MMP, PMI, and acrosome integrity) and viability after freezing-thawing (Table 2B). 

**Figure 8:**
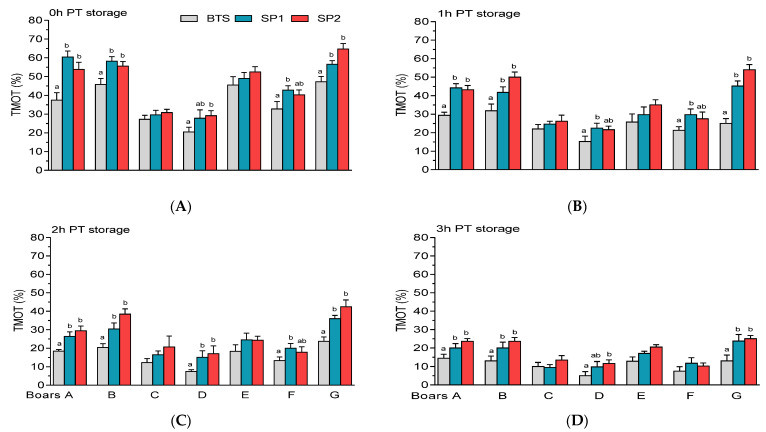
Post-thaw (PT) total motility (TMOT) of spermatozoa held in Beltsville Thawing Solution (BTS) and seminal plasma (SP) of fraction 1 (SP1) and fraction 2 (SP2) of boar ejaculates at different storage periods. (**A**) 0 h, (**B**) 1 h, (**C**) 2 h, and (**D**) 3 h PT storage. Values are expressed as the means (± SEM) of four to five ejaculates from seven boars. Values with different letters (a,b) within treatment are significant at *p* < 0.05.

Significantly higher (*p* < 0.05) PT sperm TMOT were observed in samples treated with SP1 or SP2 compared with BTS-treated samples of Boars A, B, F, and G, regardless of the PT storage period (Figure 8A–D).

**Figure 9:**
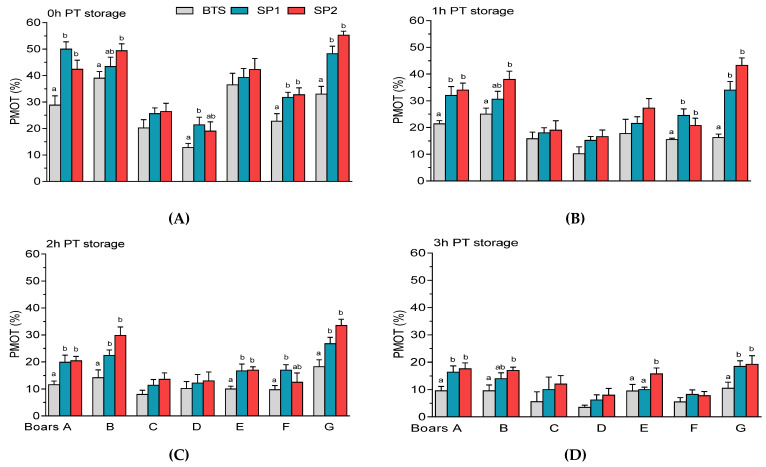
Post-thaw (PT) progressive motility (PMOT) of spermatozoa held in Beltsville Thawing Solution (BTS) and seminal plasma (SP) of fraction 1 (SP1) and fraction 2 (SP2) of boar ejaculates at different storage periods. (**A**) 0 h, (**B**) 1h, (**C**) 2 h, and (**D**) 3 h PT storage. Values are expressed as the means (± SEM) of four to five ejaculates from seven boars. Values with different letters (a,b) within treatment are significant at *p* < 0.05.

Significantly higher (*p* < 0.05) PMOT was observed in FT samples treated with SP1 and SP2 of boars A and G at different storage periods (Figure 9A–D). It was found that SP2 of boar B showed higher (*p* < 0.05) PMOT at different PT storage periods (Figure 9A–D) whereas SP2-treated samples of boar E exhibited higher (*p* < 0.05) PMOT at 2 h and 3 h PT storage (Figure 9B and Figure 9C, respectively).

**Figure 10:**
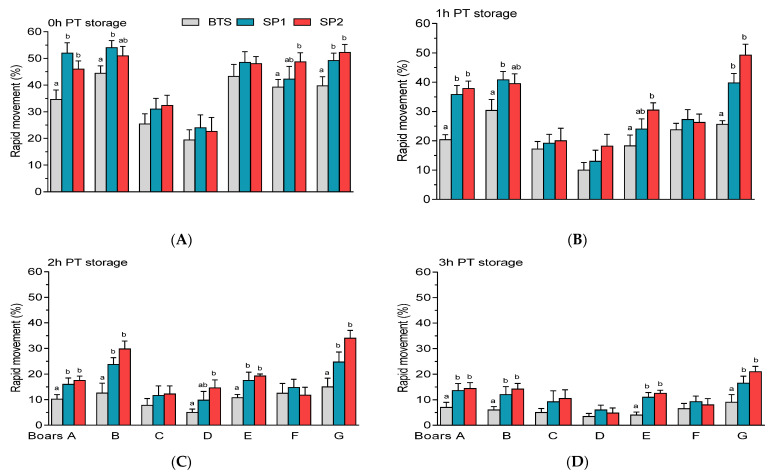
Post-thaw (PT) rapid movement of spermatozoa held in Beltsville Thawing Solution (BTS) and seminal plasma (SP) of fraction 1 (SP1) and fraction 2 (SP2) of boar ejaculates at different storage periods. (**A**) 0 h, (**B**) 1 h, (**C**) 2 h, and (**D**) 3 h PT storage. Values are expressed as the means (± SEM) of four to five ejaculates from seven boars. Values with different letters (a,b) within treatment are significant at *p* < 0.05.

Wide variations in PT rapid movement were observed among the boars, being significantly higher (*p* < 0.05) in SP1 and SP2-treated samples of boars A and G, regardless of the PT storage period (Figure 10A–D). No significant differences (*p* > 0.05) were observed between FT samples treated with SP1 and SP2, with respect to motility characteristics throughout the experiments.

**Figure 11:**
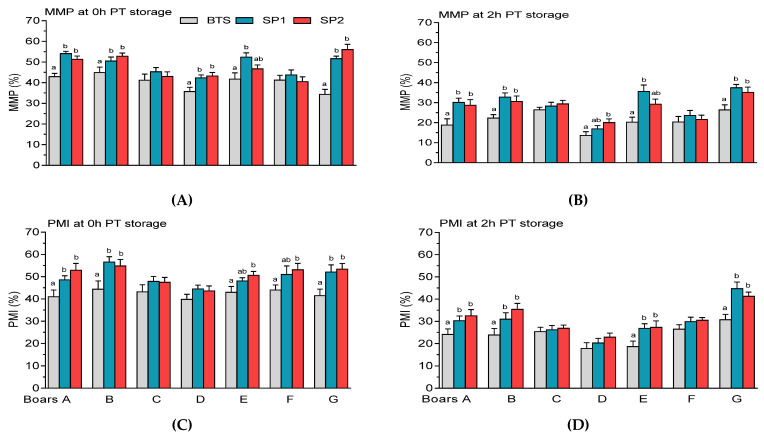
Post-thaw (PT) mitochondrial membrane potential (MMP) and plasma membrane integrity (PMI) of spermatozoa held in Beltsville Thawing Solution (BTS) and seminal plasma (SP) of fraction 1 (SP1) and fraction 2 (SP2) of boar ejaculates at different storage periods. (**A**) 0 h and (**B**) 2 h PT storage for MMP, and (**C**) 0 h and (**D**) 2 h PT storage for PMI. Values are expressed as the means (± SEM) of four to five ejaculates from seven boars. Values with different letters (a,b) within treatment are significant at *p* < 0.05.

It was observed that FT spermatozoa treated with SP1 or SP2 from boars A, B, and G exhibited higher (*p* < 0.05) MMP than those treated with the BTS extender at 0 h (Figure 11A) and 2 h PT storage (Figure 11B). Similar to post-thaw MMP, FT samples treated with SP1 or SP2 from boars A, B, and G showed markedly higher (*p* < 0.05 PMI at 0 h (Figure 11C) and 2 h PT storage (Figure 11D). SP2-treated spermatozoa of boars E and F showed higher (*p* < 0.05) PMI (Figure 11C). Prolonged PT storage of BTS-treated spermatozoa showed a marked reduction (*p* < 0.05) in PMI compared with either samples treated with SP1 or SP1 of boar E (Figure 11D).

**Figure 12:**
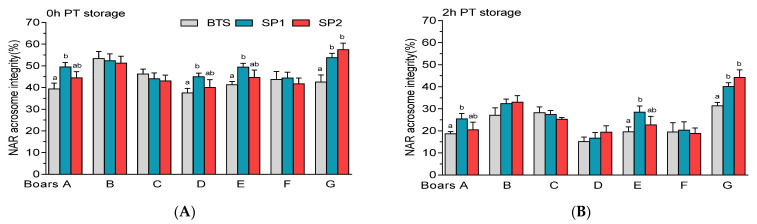
Post-thaw (PT) normal apical ridge (NAR) acrosome integrity of spermatozoa held in Beltsville Thawing Solution (BTS) and seminal plasma (SP) of fraction 1 (SP1) and fraction 2 (SP2) of boar ejaculates at different storage periods. (**A**) 0 h and (**B**) 2 h PT storage. Values are expressed as the means (± SEM) of four to five ejaculates from seven boars. Values with different letters (a,b) within treatment are significant at *p* < 0.05.

Samples treated with SP1 or SP2 from boar G exhibited higher (*p* < 0.05) percentage of FT spermatozoa with NAR acrosome integrity at 0 h (Figure 12A) and 2 h PT storage (Figure 12B). In addition, the percentages of FT spermatozoa with NAR acrosome integrity were higher (*p* < 0.05) in SP1-treated samples compared with BTS for boar A or E at 0 h (Figure 12A) or 2 h PT storage (Figure 12B).

**Figure 13:**
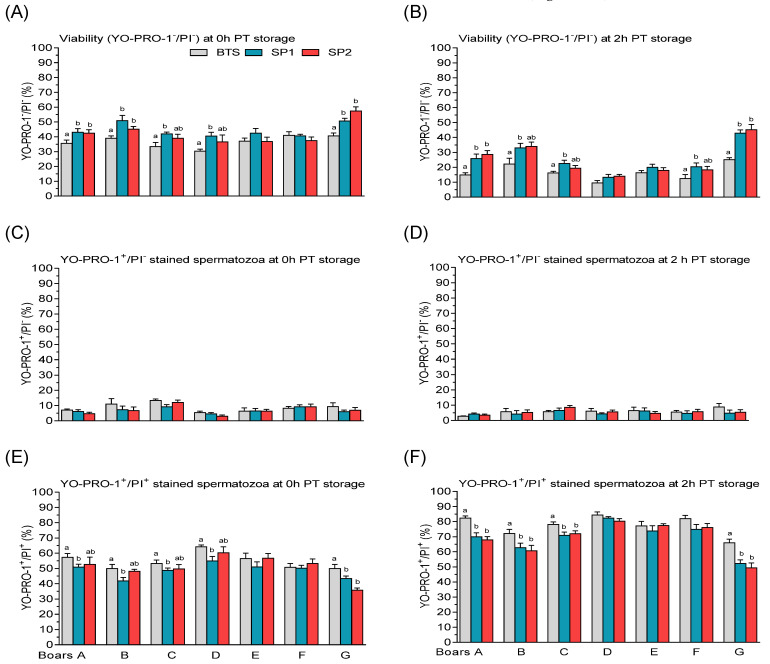
Post-thaw (PT) percentage of viable (YO-PRO-1^−^/PI^−^), plasma membrane apoptotic (YO-PRO-1^+^/PI^−^), and dead spermatozoa (YO-PRO-1^+^/PI^+^) following storage in Beltsville Thawing Solution (BTS) and fractionated seminal plasma (SP1 and SP2) of boar ejaculates at different periods. (**A**) 0 h and (**B**) 2h PT storage for YO-PRO-1^−^/PI^−^ stained spermatozoa, (**C**) 0 h and (**D**) 2 h PT storage for YO-PRO-1^+^/PI^−^ stained spermatozoa, and (**E**) 0 h and (**F**) 2 h PT storage for YO-PRO-1^+^/PI^+^ stained spermatozoa. Values are expressed as the means (± SEM) of four to five ejaculates from seven boars. Values with different letters (a,b) within treatment are significant at *p* < 0.05.

Most of the boars showed reduced (*p* < 0.05) viability (YO-PRO-1^−^/PI^−^) at 0 h (Figure 13A) and 2 h PT storage (Figure 13B). Even though there were marked (*p* < 0.05) variations among the boars (Table 2B), no significant (*p* > 0.05) differences in the percentages of frozen–thawed with plasma membrane apoptotic-like changes (YO-PRO-1^+^/PI^−^) were observed among the treated groups (Figure 13C,D). In all the boars, except for boars E and F, BTS-treated samples exhibited higher (*p* < 0.05) proportions of dead FT spermatozoa (YO-PRO-1^+^/PI^+^) compared with samples supplemented with either SP1 or SP2 at 0 h PT storage (Figure 13E). Prolonged storage of BTS-treated samples caused a significant increase (*p* < 0.05 in the percentages of dead FT spermatozoa compared with samples treated with SP1 or SP2 from boars A, B, C, and G (Figure 13F).

## 4. Discussion

In our recent studies, we demonstrated that chromatographic fractionation of boar SP could yield different protein fractions that could be incorporated in the freezing protocol to improve the quality of PT semen [27,28]. The chromatographic separation and electrophoretic analysis of SP were described in our recent paper [27]. In this study, we provide the representative profiles of the protein composition of the fractionated SP obtained by gel filtration chromatography, as described in our previous study [27]. Using label-free quantitative LC–MS/MS analysis, we detected approximately 120 proteins in the fractionated SP, which are related to various biological processes, such as cell motility, cellular process, regulation of biological process, signal transduction, and production of molecular mediator of immune response. Furthermore, GO analysis shows that sperm membrane- and cytoskeletal-associated proteins were found in SP1 and SP2, particularly in the former. A possible reason for such phenomenon could be to the interactions of these proteins with the epididymal fluids during maturation and at ejaculation. Evidence has shown that a wide variety of sperm proteins are expressed in high levels in the bovine, goat, boar, and canine SP [11,12,26,43]. It was suggested that proteins occurring in both sperm and SP proteome could be due to non-specific aggregation of spermatozoa and SP proteins or might indicate an active and/or protein transport mechanism between the sperm cells and SP [43,44]. In different animal species, it has been confirmed that the SP comprises a diverse set of protein components, which bind to spermatozoa and affect their membrane structure and fertilizing ability [8,9,11,12,26]. Moreover, SP proteins also affect the physiology of the female reproductive tract, influencing the immune and inflammatory response processes, and embryo development [9,45,46]. We suggest that components of the fractionated SP are involved in important events related to reproduction and have relevance in the female reproductive tract during fertilization and embryo development.

The predominant biological function of SP1 was associated with cell motility, a functional attribute of spermatozoa [6,7,19,27,37]. We suggest that the wide variations in the motility of FT spermatozoa, observed following PT storage, could be due to differences in the composition of the SP fraction from each boar; however, the mechanisms involved in the interactions of the SP proteins with the sperm membrane are still not fully understood. Differences in the amounts of total SP protein content and the different types of SP proteins among boar ejaculate fractions have been reported in other studies [14,15,17]. Among the KEGG pathways detected in SP1, the apelin signaling pathway, comprising GNA13, MEF2D, SPHK2, and MEF2D proteins, was the most dominant one. Even though it was reported that the apelin signaling pathway is implicated in the regulation of steroidogenesis, cell proliferation, and apoptosis in gonads, and has an inhibitory action on several kinase pathways [47], it is unclear about the significance of the signaling transduction pathway in sperm function. More recently, it was demonstrated that SP infusions prior to AI resulted in the upregulation of genes associated with the apelin signaling pathway, suggesting its signal transduction role in embryo development [46]. It is likely that the GNA13 protein binds to the spermatozoa, and its higher expression (7- to 30-fold) compared with the other three abundant proteins (MEF2D, SPHK2, and MEF2C) indicates its relevance in the sperm functions. It is noteworthy that GNA13 is a member of the G binding protein α subunit family that has been detected in the testes and epididymis, and could play a role in gonad development and sperm maturation [48]. Furthermore, GNA13 has been detected in the sperm membrane, and is suggested to be involved in the regulation of several sperm functions, such as motility, acrosome reaction, and fusion with the oocyte [49]. Moreover, the cGMP-PKG signaling pathway is suggested to promote hyperactivation, and is implicated in several sperm signaling pathways functions, such as capacitation, acrosome reaction, and sperm–egg interaction [50]. However, we are unable to explain the impact of this pathway on the cryo-survival of boar spermatozoa. Analysis shows that GNA13, SPHK2, and MEF2C proteins are related to a GO term, “regulation of cell motility” (GO:2000145), which might suggest an important regulatory role of these proteins in sperm motility during PT storage. Interestingly, the protein–protein network interaction shows that NUP107, NUP210, GNA13, MEF2C, and several other proteins are involved in the HSF1-mediated shock response pathway, suggesting the contributions of these proteins to sperm protection. It may be worth it to further investigate the biological functions of these SP-derived proteins in sperm cryo-survival.

It is noteworthy that N-glycan biosynthesis pathway is associated with fertilization-related events [51] and oviductal sperm colonization [52], however, its role in sperm function following cryopreservation has not been elucidated as yet. Of interest is HEXB, which is found in the acrosome and plasma membrane of boar spermatozoa and is suggested to be implicated in the sperm–egg fertilization events [51]. Higher levels of HEXB activity in boar SP were associated with reduced sperm cryo-tolerance, suggesting the role of the protein as a sperm freezability marker [51]. However, in the current study, we are unable to explain whether HEXB activity was responsible for the poor PT semen quality.

Remarkably, the predominant BP detected for SP2 proteins was related to the regulation of biological process, which is represented by several proteins, such as cathepsins and TRIM24. Moreover, enrichment KEGG pathways of SP2 proteins were associated with lysosome and apoptosis representing by cathepsins (CTSH and CTSB) and NPC2. Besides PSP-1 and PSP-II, CTSH was the third most abundant protein in SP2. Cathepsins are proteases [53] and represented the predominant protein class in SP2. Functional analysis shows that cathepsins are annotated to GO terms, such as “cell death” (GO:0008219), “apoptotic process” (GO:0006915) and proteolysis” (GO:0006508), suggesting their involvement in the sperm survival during PT storage. Moreover, cathepsins are involved in the remodeling of sperm membrane during epididymal maturation and in the SP, as they could interact with the membrane structure of spermatozoa and exert a protective role by suppressing sperm-related apoptotic changes via an intrinsic pathway [10,11,13,53]. In a previous study, it was reported that CTSB levels were negatively correlated with ram sperm resistance to freezing [54], whereas in poor quality ejaculates high CTSB levels were associated with reduced progressive motility and increased sperm morphological defects, suggesting the possible role of the protein as a marker for semen quality [53]. The protein–protein network interaction, representing the MHC class II antigen presentation pathway, confirms that cathepsins interact with a wide variety of proteins that are implicated in the immune-related pathway [10,53,54]. Similar to cathepsins, high abundance of TRIM24 was found in SP2, and its biological function was related to the apoptotic pathway and cell death, reaffirming the finding of a previous study indicating that TRIM24 might be implicated in p53-dependent apoptosis and DNA damage response [55]. Surprisingly, no marked differences were detected among the treated groups with respect to the proportions of FT spermatozoa with apoptotic-like changes. However, reduced motility, membrane integrity, and viability of FT spermatozoa were concomitant with an increase in the proportions of dead spermatozoa following PT storage, suggesting that CTSH and TRIM24 abundance could compromise the sperm survival. We suggest that further investigations are needed to explain the functional relevance of CTSH and TRIM24 in sperm cryo-survival.

Notably, NPC2 protein was less abundant in SP2 compared with CTSH. Moreover, NPC2, a high-affinity cholesterol-binding protein, is secreted in different tissues of the reproductive organs [5,56,57], and has been described to be among the most abundant secreted proteins in bovine epididymis [58]. The NPC2 protein is suggested to participate in cholesterol efflux from the spermatozoa, during epididymal sperm maturation and in the SP, and could act as a membrane reorganization factor in a capacitation-independent process [57,59]. Furthermore, the relative higher levels of two isoforms of NPC2 protein (16 kDa and 19 kDa) found in the SP of boars with poor semen freezability (PSF) than in those with good semen freezability (GSF) suggest that the protein could be useful to predict sperm cryo-tolerance [4,5]. However, the protein composition of the fractionated SP of each boar was not analyzed individually, so it is difficult to explain whether NPC2 abundance was associated with reduced PT semen quality. We suggest that further studies are needed to clarify these findings.

One of the main objectives of this study was to assess the effects of fractionated SP supplementation to FT boar semen on motility characteristics, membrane integrity, and viability of spermatozoa from individual boars. We hypothesized that the components of the fractionated SP could minimize the storage-induced damage to FT spermatozoa following prolonged PT storage. Our results clearly indicate that FN1 is the most abundant protein in SP1. Particularly in the boar, the most abundant proteins in the SP are FN1 protein and spermadhesins, which play key roles in the male and female reproductive tracts [8,9,13,60]. Evidence has shown that the interaction of FN1 protein with spermatozoa resulted in reduced oxidative stress in the sperm midpiece and tail regions, contributing to improved motility [61]. Furthermore, FN1 protein is suggested to be a potential marker for the freezability of boar semen, and its expression is correlated with sperm motility [62]. We suggest that the high abundance of FN1 protein could partially explain the improved protective effects on sperm membrane following PT storage, particularly in boars A, B, and G. In addition, the high abundance of KIF15, ANGPTL1, and SOCS5 in SP1 may indicate the functional significance of these proteins in sperm function. It is noteworthy that GO analysis shows that KIF15, a binding protein, is related to “microtubule-based movement” (GO:0007018) and is implicated in spermatogenesis [63], reaffirming the role of the protein in sperm motility during PT storage. Furthermore, kinesin-like proteins in human SP are suggested to play a role in organelle transport [64], whereas their role in goat SP has not been elucidated [12]. Likewise, the biological functions of ANGPTLI and SOCS5 proteins are related to the GO term “regulation of cell motility”, suggesting their contributions to sperm cryo-survival. Furthermore, the SOCS family members are regulatory proteins that are responsible for mediating the signals of cytokine-induced immune responses [65], suggesting a potential role of these proteins in the female reproductive tract. Interestingly, the predominance protein class in SP1 was transporter, representing by the solute carrier (SLC) mediated transmembrane transporters, such as SLC6A1, SLC22A15, and SLC16A1. The SLC super family is implicated in the transport of a wide variety of substances across cell membranes [66] and are suggested to be structural proteins [8]. The precise role of SLC proteins in SP is still unclear; however, these proteins are suggested to be involved in the regulation of pH [67]. Similar to SLC proteins, FAM149A is a structural protein previously detected in boar SP [8]. It seems that SLC and FAM149A proteins could interact with the sperm membranes; however, the possible mechanism by which these proteins could act to influence sperm cryo-survival remains to be investigated.

Other highly abundant proteins detected in SP1 are UBQLNL, FCGBP, EPHB1, and AKNA. Notably, the testis ubiquitin-like protein (UBQLNL) is involved in spermatogenesis [68]. Analysis shows that UBQLNL is related to GO terms, such as “cellular response to hypoxia” (GO:0071456), and “positive regulation of protein ubiquitination” (GO:0031398), suggesting the role of the protein in various cellular regulatory processes. However, the mechanisms by which UBQLNL could contribute to sperm survival remains to be investigated. Studies have shown that immunoglobulins bind to antigens of the sperm surface and promote agglutination and immobilization, resulting in reduced cervical mucus penetration and compromised acrosome reaction and gamete interaction [61]. Moreover, it was confirmed that high expression of FCGBP in boar SP was associated with poor freezability ejaculates [69]. In the current study, it is possible that FCGBP abundance could be responsible for the poor PT sperm survival observed in the boars. Although not much is known about the role of EPHB1, the protein is annotated to several GO term, such as “regulation of cell motility”, “response to stimulus” (GO:0050896), and “phosphorylation” (GO:0016310), suggesting its contribution to the sperm functionality. GO analysis shows that AKNA protein are related to “signal transduction” (GO:0007165) and “regulation of response to stress” (GO:0080134), indicating its regulatory role in sperm function. These observations deserve further research to clarify the role of EPHB1 and AKNA proteins in sperm function, and how these proteins could interact with the sperm membrane to improve sperm cryo-survival. It is worth pointing out that, besides protein components, SP comprises growth factors, cytokines, and transcription factors that are important for protection of spermatozoa and maintenance of their functions during transit in the male and female reproductive tracts [8,9,11,13].

Additionally, SP2 was characterized by the high abundance of spermadhesins, (PSP-I and PSP-II), CTSH, and TRIM24. Besides, PSP-I and PSP-II, other proteins, such as AWN-1 and AQN-3 spermadhesins, were abundant in SP2. LC–MS/MS analysis shows that heparin non-binding PSP-I is the predominant spermadhesin in SP1 followed by PSP-II. In the current study, the presence of spermadhesins could be responsible, at least in part, for the improved PT sperm quality. Spermadhesins are mainly implicated in different types of interactions with spermatozoa, such as the remodeling of the sperm membranes through their binding activity, and it is noteworthy that the beneficial effects of these sperm-coating proteins in fertilization, liquid storage, or cryopreservation of semen have been extensively covered in numerous studies [8,9,10,13,15,19,25,60,61]. Of particular interest is the PSP-I/PSP-II heterodimer because of its functional attributes observed during reproductive technologies, such as semen preservation. It is well known that SP heparin-binding proteins (AWN-1, AQN-3, and AQN-1) play an important role in the stability of the membrane overlying the sperm acrosomal region and are involved in the formation of the oviduct reservoir and gamete interaction [9,13,25,61]. Besides spermadhesins, cathepsins, NPC2, and TRIM24, analyses show that the biological functions of most of the abundant proteins are related to several GO terms, such as “signal transduction” (GO:0007165), “cellular process” (GO:0009987), and “macromolecule metabolic process” (GO:0043170), suggesting their multifunctional roles in different reproductive processes. Some of these proteins are related to proteinase inhibitors, collagens, cytoskeleton organization, and immune response. We suggest that the role of these proteins in sperm functionality needs to be further explored.

Altogether, the findings of the present study show that sperm motility characteristics and membrane integrity, represented by MMP, PMI, acrosome integrity, and viability, were consistently higher in both SP1 and SP2 compared with BTS following PT storage, particularly in boars A, B, and G. We suggest that the presence of proteins in SP1 or SP2, with high and low abundance, exerted better protective effects on the membrane structures of spermatozoa, resulting in their improved survival following PT storage. Furthermore, it is plausible to hypothesize that the interactions of the proteins in either SP1 or SP2 with components of the BTS extender might have beneficial effects on the sperm membrane and contribute to improved PT sperm quality. Moreover, it was demonstrated that the supplementation of 10% SP to the thawing medium significantly improved PT semen quality and the fertilization ability of FT spermatozoa from boars with poor freezability ejaculates [16]. In another study, it was reported that supplementation of 50% SP to the thawing medium improved PT sperm function and fertility [20]; however, in another study, PT supplementation of 50% SP had negative effects on sperm function [70]. We suggest that such disparity in the SP-based studies on PT semen quality might be due to several factors, namely the final concentrations of SP, composition and source of the SP. It is evident that the loss of motility and membrane integrity of boar spermatozoa following PT storage is mainly due to the increased oxidative stress, resulting in excessive production of reactive oxygen species (ROS) [6,18,24,70].

Using label-free quantitative LC–MS/MS analysis, we identified a plethora of proteins with high and low abundance in the fractionated SP that might be involved in the sperm membrane modifications during PT storage. Interestingly, there were no marked differences in sperm quality between the fractionated SP, even though SP1 is compositionally different from SP2. It is tempting to speculate that the improved sperm quality observed following PT storage was not due to the presence of a single protein in SP1 or SP2, but rather to the concerted action of a diverse cohort of proteins in either fractionated SP, which exerted beneficial effects on the sperm’s structural and functional membranes. Further investigations are required to confirm the relevance of high and low abundance proteins in boar SP in sperm cryo-tolerance, which are crucial to unravel their biological roles in semen cryopreservation.

## Figures and Tables

**Table 1:** Sperm quality characteristics in fresh boar ejaculates.

Sperm Parameters (%)	Means ± SEM (*n* = 32)
Total motility (TMOT)	86.97 ± 1.06
Progressive motility (PMOT)	59.52 ± 1.59
Rapid movement (%)	60.06 ± 2.70
Mitochondrial membrane potential (MMP)	85.80 ± 0.35
Plasma membrane integrity (PMI)	86.98 ± 0.31
Acrosome integrity (%)	88.94 ± 0.37
Viability (YO-PRO-1^−^/PI^−^)	86.16 ± 0.66
Plasma membrane apoptotic-like changes (YO-PRO-1^+^/PI^−^)	5.81 ± 0.35
Dead spermatozoa (YO-PRO-1^+^/PI^+^)	8.03 ± 0.41

**Table 2:** ANOVA sources of variations in post-thaw (PT) quality characteristics of boar spermatozoa. (**A**) Sperm motility parameters. (**B**) Membrane integrity and viability parameters.

Sperm	Boar	Treatment	PT Storage	Boar × Treatment	Boar × PT Storage
Parameters	*p*-Value	*p*-Value	*p*-Value	*p*-Value	*p*-Value
(A)					
Total motility (TMOT)	0.001	0.001	0.001	0.009	0.001
Progressive motility (PMOT)	0.001	0.001	0.001	0.008	0.001
Rapid movement	0.001	0.001	0.001	n.s	0.003
**(B)**					
Mitochondrial membrane potential (MMP)	0.001	0.001	0.001	0.001	0.001
Plasma membrane integrity (PMI)	0.001	0.001	0.001	*n.s*	0.001
Acrosome integrity	0.001	0.001	0.001	0.019	0.001
Viability (YO-PRO-1^−^/PI^−^)	0.001	0.001	0.001	0.001	0.001
Plasma membrane apoptotic-like changes (YO-PRO-1^+^/PI^−^)	0.001	n.s	n.s	n.s	0.043
Dead spermatozoa (YO-PRO-1^+^/PI^+^)	0.00.1	0.001	0.001	0.017	0.001

Factorial design; boar (7) × treatment (3) × PT storage (4); significant at *p* < 0.05; n.s—non-significant. Factorial design; boar (7) × treatment (3) × PT storage (2); significant at *p* < 0.05; n.s—non-significant.

## Supplementary Materials

The following are available online at https://www.mdpi.com/article/10.3390/genes12101574/s1. Supplementary Table S1. Identification and quantification of protein data of fractionated seminal plasma (SP) of boar ejaculates, using MaxQuant workflow. Supplementary Table S2. KEGG and Reactome (REAC) pathways of differentially abundant proteins in fractionated seminal plasma (SP1) of boar ejaculates. Supplementary Table S3. KEGG and Reactome (REAC) pathway of differentially abundant proteins in fractionated seminal plasma (SP2) of boar ejaculates.
